# Evaluation of *Legionella* spp. Colonization in Residential Buildings Having Solar Thermal System for Hot Water Production

**DOI:** 10.3390/ijerph17197050

**Published:** 2020-09-26

**Authors:** Michele Totaro, Anna Laura Costa, Lorenzo Frendo, Sara Profeti, Beatrice Casini, Antonio Gallo, Gaetano Privitera, Angelo Baggiani

**Affiliations:** 1 Department of Translational Research N.T.M.S, University of Pisa, 56123 Pisa, Italy; micheleto@hotmail.it (M.T.); anna.costa@med.unipi.it (A.L.C.); lorenzo.frendo@hotmail.com (L.F.); profeti.sara@gmail.com (S.P.); beatrice.casini@med.unipi.it (B.C.); gaetano.privitera@med.unipi.it (G.P.); 2 Division of Public Health and Nutrition, Area of Pisa, Azienda USL Toscana Nord Ovest, 56123 Pisa, Italy; antonio.gallo@uslnordovest.toscana.it

**Keywords:** *Legionella* spp., residential buildings, waterborne pathogens, solar thermal panels

## Abstract

Despite an increase of literature data on *Legionella* spp. presence in private water systems, epidemiological reports assert a continuing high incidence of Legionnaires’ disease infection in Italy. In this study, we report a survey on *Legionella* spp. colonization in 58 buildings with solar thermal systems for hot water production (TB). In all buildings, *Legionella* spp. presence was enumerated in hot and cold water samples. Microbiological potability standards of cold water were also evaluated. *Legionella* spp. was detected in 40% of the buildings. Moreover, we detected correlations between the count of *Legionella* spp. and the presence of the optimal temperature for the microorganism growth (less than 40 °C). Our results showed that cold water was free from microbiological hazards, but *Legionella* spp., was detected when the mean cold water temperature was 19.1 ± 2.2 °C. This may considered close to the suboptimal value for the *Legionella* growth (more then 20 °C). In conclusion, we observed the presence of a Legionnaires’ disease risk and the need of some strategies aimed to reduce it, such as the application of training programs for all the workers involved in water systems maintenance.

## 1. Introduction

The importance of opportunistic waterborne pathogens has been increasing in recent years. *Legionella* spp. has been identified in European surveillance reports as the most common cause of waterborne infection outbreaks [1]. *Legionella* spp. is widely present in the environment and may colonize water systems. This occurrence is mostly present in old buildings, corroded pipelines and dead leg branches, allowing the biofilm growth in sites where disinfectants are ineffective against microorganisms [2,3,4]. Legionnaires’ disease is acquired by inhalation and aspiration of droplets or aerosols carrying *Legionella* spp. Chronic diseases and immunodepression are predisposing factors for the development of the disease, mostly caused by *Legionella pneumophila* serogroup 1 [5]. The surveillance of Legionnaires’ disease is conducted in Europe, by the European Center for Disease Prevention and Control and in Italy by the Italian National Institute of Health.

The latest Italian data from 2018, document 2964 cases of Legionnaires’ disease in the Italian population, corresponding to a notification rate of 48.9 cases per million inhabitants. However, it must be noted that 2018 showed an increase of 32% in cases, as compared with the previous year. Of the 2964 notified cases, 101 (3%) were hospital acquired, while 2497 (84%) were community-acquired infections, highlighting the prevalence of *Legionella* spp. colonization in community environment [6].

During the last three years, an increase of literature data on *Legionella* spp. presence in water networks of residential buildings has been observed [7,8,9,10,11]. Moreover, both an Italian guideline for Legionnaires’ disease control in hospital and community settings, and the Drinking Water Directive identify the responsibilities for water quality control in private residential buildings [12,13].

In a three-year survey conducted from 2014 to 2017 on 220 residential buildings located in the Pisa District (Italy), we found the colonization of *Legionella* spp. in 26% of the hot water systems [9]. Following these published data, the purpose of this new study is the evaluation of *Legionella* spp. colonization in hot and cold water systems in private domestic using solar thermal panels.

## 2. Materials and Methods

### 2.1. Setting and Solar Thermal Systems Description

The survey was conducted from April 2017 to April 2019 on 58 domestic apartments with a solar thermal system for hot water production (TB). The buildings located in Pisa (Italy) were mainly of small sizes with ranges between four and fifteen flats and between one and five floors. In each building, a water storage tank receives municipal or well water in order to feed the entire water system.

Inspection visits were conducted to verify the adequate functioning of the thermal and water power plants. Inspections were conducted following a checklist organized with the name, address of the building, water disinfection system, periodicity and type of water system maintenance and cleaning, water supplying and number of floor and apartments, as described elsewhere [9].

All solar thermal systems have a natural circulation plants. These devices use commercial solar thermal collectors arranged in a self-compensating natural circulation system. They catch the solar energy for hot water production, which is stored in a special tank. Therefore, hot water may be introduced in domestic water plant. Overall, the investigated thermal systems consist of a panel structure (glass with a steel heat absorber), a water storage tank (from 40 to 60 L/m^2^ of panel surface), hydraulic connections and some mounting brackets.

Each building presented a different solar thermal system for hot water production.

### 2.2. Sampling and Laboratory Tests

**Figure 1:**
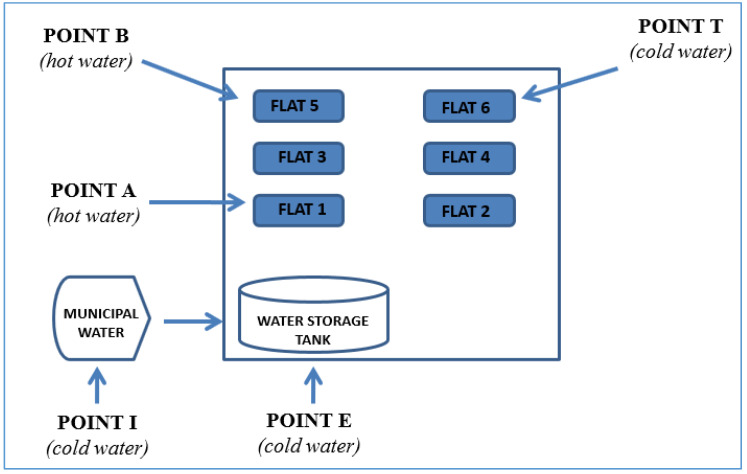
Schematic figure of the hot and cold sampling points in each building.

From each water system, three cold water samples were taken: (1) one at the inlet from the aqueduct (municipal water, outside the building) (Point I), (2) one at the exit from the water storage tank (inside the building) (point E) and (3) one at the most remote tap from the tank (point T) (Figure 1). A total of 174 cold water samples were analyzed for the determination of the microbiological potability requirements, as stated by the Council Directive 98/83/EC [12].

Total microbial count at 22 °C and at 37 °C, coliform and Enterococci counts were determined according to the Council Directive 98/83/EC, as described in our previous study [9].

The presence of *Legionella* spp. was investigated in two hot water sampling taps, located at the first floor of the building (Point A) and on the last floor (Point B). The presence of *Legionella* spp. was also investigated in cold water collected at the most remote tap (point T) (Figure 1).

In total, 116 hot water samples and 58 cold water samples were collected for *Legionella* spp. detection, as suggested by the Italian guidelines for legionellosis control (Italian National Institute of Health, 2015). Samples were tested for *Legionella* spp. presence according to ISO 11,731:2017 [14] as described elsewhere [9]. Confirmed *Legionella* spp. colonies were tested for species and serogroup by polyvalent agglutination latex test (*Legionella* latex test—Oxoid, UK).

Water temperature and total chlorine concentration were determined in all the water samples, while pH and conductivity values were measured only in water samples collected for *Legionella* spp. detection.

### 2.3. Correlation Tests

Correlation tests were conducted, and Pearson’s coefficients were calculated with the aim to analyze the correlations between physical–chemical parameters of the samples (temperature, chlorine concentration and conductivity) and the presence of *Legionella* spp. Confidence levels of 95% were defined for the statistical tests. Therefore, we considered the following ranges of values: 0.7–1 (strong correlation), 0.3–0.7 (moderate correlation), 0–0.3 (weak correlation).

## 3. Results

### 3.1. Inspections and Physical–Chemical Results

All the 58 central water supplies had a water storage tank. Forty-six of 58 (79%) water plumbing systems distributed municipal water, while 12 of 58 (21%) of TB are fed by wells. Only municipal water was chlorinated before entering in TB system (total chlorine mean of 0.047 ± 0.007 mg/L), while the suggested values is 0.2 mg/L [11]. No buildings had either softener or an adequate continuous or periodic disinfection method. No TBs presented an overheating risk, which was evaluated by the temperature limit value shown in technical certificates (from 75 to 85 °C). After reaching this limit, panels increased the reflection percentage, blocking the infrared radiation, avoiding overheating. Therefore, all the cold water (19.1 ± 2.2 °C) and hot water (33.8 ± 9.4 °C) samples from TB were not chlorinated with an adequate continuous disinfection method. All central water supplies had a boiler for water storage, heated by solar thermal systems. Physical–chemical data, measured in hot water samples, showed pH values ranging from 5.6 to 7.3 (mean 6.7 ± 0.8) and conductivity values between 935 to 1156 µS/cm^2^ (mean 1101 ± 125 µS/cm^2^). Maintenance programs were absent in all the TB.

All physical–chemical value regarding the cold and hot water samples, collected at different points of use, are shown in Table 1.

### 3.2. Microbiological Results

A variability in microbial growth at 22 and 37 °C was observed among the cold water samples. Total microbial counts at 22 and 37 °C were between 1 and 300 CFU/mL. Bacterial counts higher than 100 CFU/mL were detected in 36% (63 of 174) of the samples, mostly at Point E and Point T. Overall, all samples resulted free from microbiological hazards. In fact, coliforms and enterococci bacteria were not detected in any water sample.

*Legionella* spp. was detected in 46 of the 116 (40%) hot water samples and in seven of the 58 (12%) cold water samples examined. Totally, 53 of the 174 (30%) water samples resulted positive for *Legionella* spp.

Overall, 23 of the 58 (40%) examined buildings had at least one sample positive for *Legionella* spp. In all the seven buildings with the cold water system colonized by *Legionella* spp. also the hot water system resulted positive.

**Figure 2:**
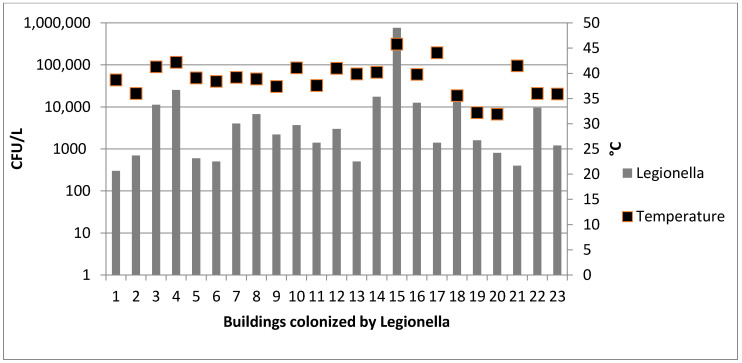
*Legionella* spp. counts and temperature values detected in 23 colonized solar thermal system for hot water production (TB).

*Legionella*-positive samples showed counts from 2 × 10^2^ to 7.6 × 10^5^ CFU/L (mean 3.8 × 10^4^ ± 1.6 × 10^4^ CFU/L) (Figure 2). *Legionella pneumophila* sg1, *Legionella pneumophila* sg2–16; and *Legionella* spp. were, respectively recovered in 10 of the 23 (44%), nine of the 23 (39%) and four of the 23 (17%) water samples.

**Figure 3:**
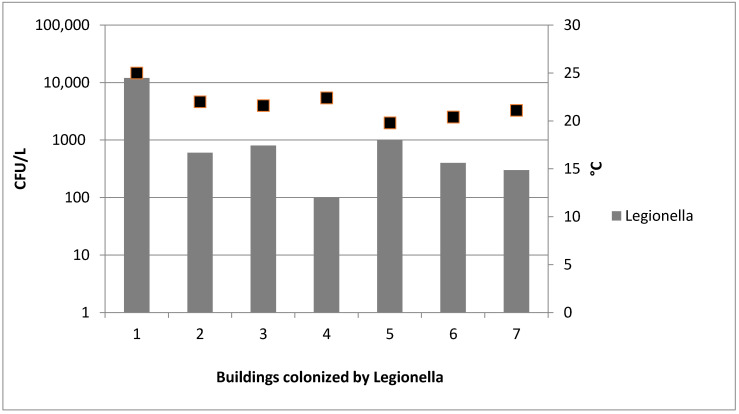
*Legionella* spp. counts and temperature values detected in seven colonized cold water systems.

Finally, *Legionella* spp. was detected in seven cold water systems with counts between 1 × 10^2^ and 1.2 × 10^4^ CFU/L (mean 2.2 × 10^3^ ± 4.4 × 10^2^ CFU/L) (Figure 3). *Legionella pneumophila* sg1, *Legionella pneumophila* sg2–16; and *Legionella* spp. were, respectively recovered in 2 of the 7 (29%), one of the seven (14%) and four of the seven (57%) water samples.

In hot water samples, a moderate correlation was detected between the *Legionella* spp. concentration and the decrease of the temperature values (r = 0.46; *p* = 0.035).

In cold water samples, statistical results showed strong correlation between the presence of *Legionella* spp. and the increase of temperature values (r = 0.82; *p* < 0.001).

## 4. Discussion

Although literature data regarding the risk of *Legionella* spp. in community settings (residential buildings, touristic and sport facilities, etc.) has increased during the last years, epidemiological reports regarding the incidence of Legionnaires’ disease in Italy and Europe have not changed during the same period [15,16,17,18].

Furthermore, in our previous studies [7,9] we highlighted the need of water safety plans applied to residential buildings, aimed to manage the underestimated *Legionella* spp. risk in these facilities.

A difference between our previous study [8] and these data concerns the type of hot water production. In this study, an higher risk of Legionnaires disease has been observed in residential buildings with solar thermal systems. In fact, we detected *Legionella* spp. in 26% of apartments in buildings provided with a centralized or independent warm water production [8]. Considering only the solar thermal systems for hot water production, the positivity percentage was higher (40%).

Moreover, we described the responsibility of the building administrators in ensuring the water hygienic control from the point of delivery by the water supplier up to the points of use.

The presence of *Legionella* spp. in 40% of all 58 investigated TB may be due to the lack of maintenance activities often highlighted during the inspections. In fact, in all the TB, we observed absence of disinfections and low temperature values (usually less then 40 °C).

Low-temperature values are due to the discontinuous activity of the storage tanks included in solar thermal systems, which may cause a progressive water heating and cooling. This activity is a risk factor for *Legionella* spp. growth, as described elsewhere [19].

Another issue evidenced by this study is the high temperature of cold water (higher than 20 °C in several cases), which may represent an optimum condition for *Legionella* spp. growth [20,21].

All these drawbacks are considered risk factors for *Legionella* spp. proliferation in water systems, as described by Italian Guidelines for Legionnaires’ disease prevention and control [13].

In fact, we detected correlations between the increase of *Legionella* spp. counts and the decrease and increase of temperature in hot and cold water samples, respectively. A strong correlation between *Legionella pneumophila* strains and high temperature values have also been observed in further studies [22], which highlights how pasteurization methods may disinfect the waters, but it may influence the persistence of thermo-tolerant *Legionella pneumophila* bacteria in viable, but non cultivable (VBNC) forms.

Moreover, we detect higher microbial counts in water samples collected at Point E and Point T. This evidence may be due to biofilm formation in pipe works and the presence of VBNC forms, as described elsewhere [23,24,25].

## 5. Conclusions

In conclusion, our work confirms *Legionella* spp. risk in the residential buildings of Pisa District (Italy).

Considering the epidemiological data regarding the incidence of Legionnaires’ disease in Italy over a two-year survey, we stress the need of water safety plans for these type of facilities, highlighting the responsibility of building administrators for appropriate hot and water hygienic control, through monitoring schemes of water systems, at least on a yearly basis.

Through this study, we can see a need for training programs for all workers involved in residential water systems management (building administrators, plumbers).

Theoretic trainings may deal with issues such as the legislative requirements from drinking water; technical maintenance of water devices; microbiological and chemical risk in pipelines; application of periodic disinfection methods; etc.

These may be the tools needed to disseminate knowledge regarding Legionnaires’ disease risks in community facilities and improve public health safety.

## Figures and Tables

**Table 1:** Building with solar thermal system for hot water production (TB).

Physical–Chemical Parameters	Hot Water Samples		Cold Water Samples		
	Point A	Point B	Point I	Point E	Point T
**Total Chlorine (mg/L)**	0	0	min: 0	0	0
			max: 0.09		
			mean: 0.047 ± 0.007		
**pH**	min: 5.6	min: 5.6	NA	NA	NA
	max: 7.1	max: 7.3			
	mean: 6.6 ± 0.7	mean: 6.7 ± 1			
**Conductivity (µS/cm^2^)**	min: 935	min: 961	NA	NA	NA
	max: 1156	max: 1140			
	mean: 1011 ± 135	mean: 1100 ± 165			
**Temperature (°C)**	min: 31.8	min: 32.2	max: 21.3	min: 18.8	min: 18.9
	max: 46	max: 45.6	max: 21.3	max: 21.4	max: 24.8
	mean: 38.8 ± 7.5	mean: 32.6 ± 9.9	mean: 18.9 ± 2.7	mean: 19.6 ± 2.1	mean: 19.9 ± 2.8

Physical–chemical values (total chlorine, pH, conductivity and temperature) parameters detected in hot and cold water sampled at different point of use (Point A, Point B, Point I, Point E and Point T) of the buildings. NA—not applied.
